# Supplementary material for: Lack of Diaph3 relaxes the spindle checkpoint causing the loss of neural progenitors
Source: Nat Commun. 2016 Nov 16;7:13509. doi: 10.1038/ncomms13509 (PMC5476800; doi:10.1038/ncomms13509)
Supplement: Supplementary Information — Supplementary Figures 1-11 and Supplementary Table 1. [file ncomms13509-s1.pdf]

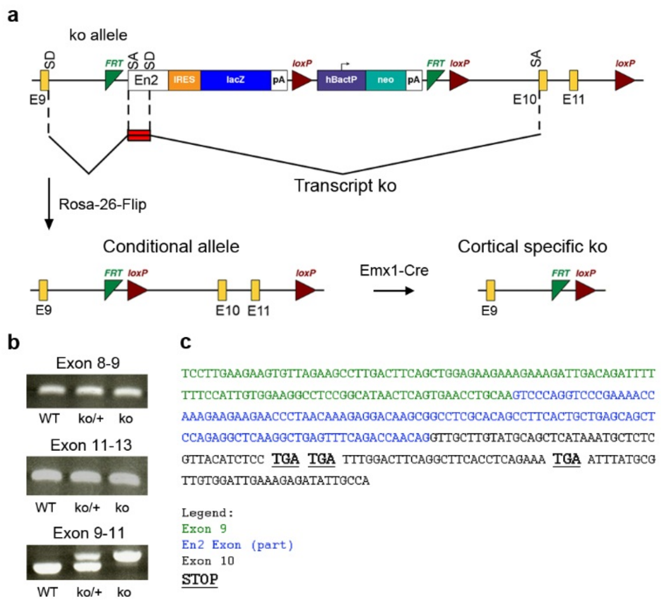

Supplementary Figure 1 Generation of *Diaph3 ko* mice.

(**a**) Scheme depicting the strategy used to generate the *ko* and conditional alleles. (**b**) RT-PCR for different regions of *Diaph3* mRNA from WT, heterozygote (*ko/+*) and mutant (*ko*) littermates showed that mRNA is present downstream of the mutation. Sequencing of the cDNA from *ko* embryo revealed a cryptic splice donor in the exon En2 resulting in premature and aberrant splicing of the cassette with insertion of 115 nucleotides (**c**, sequence in blue) between exons 9 (green) and 10 (black). A frameshift mutation was produced yielding 3 stop codons (bold underlined) in exon 10. The conditional allele was obtained by removal of the FTR-En2-IRES-LacZ-loxP-neo-FRT cassette after crossing with *ROSA26-Flp* mice. Exons 10 and 11, flanked by two loxP sites, were excised in dorsal telencephalon by crosses with *Emx1-Cre* mice.

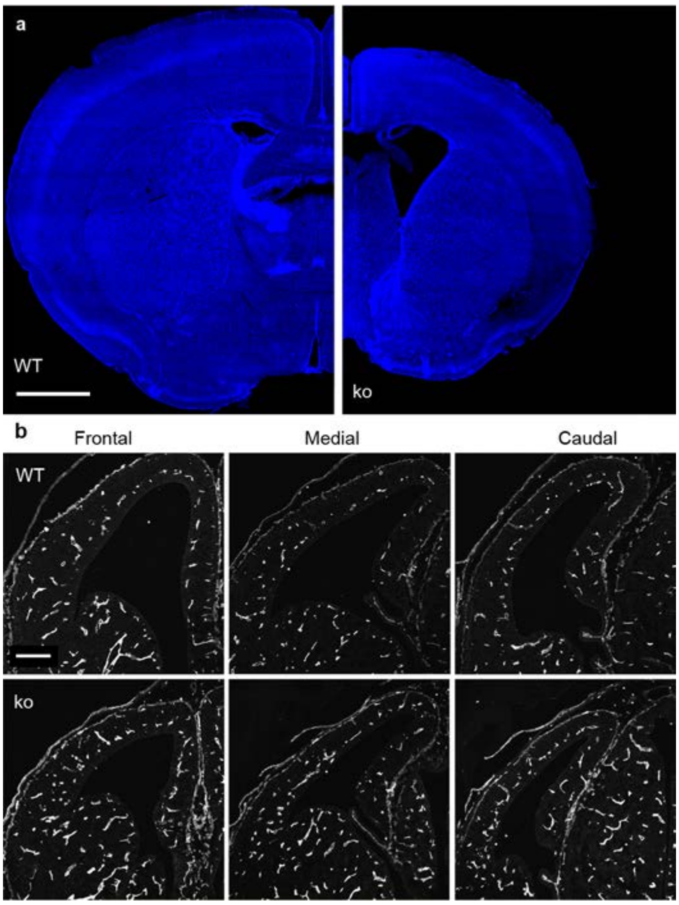

Supplementary Figure 2 Phenotype of the adult brain.

(**a**) DAPI-stained coronal sections from P25 brains of WT and *Diaph3 ko* littermates. Microcephaly and hydrocephalus are obvious in the mutant. (**b**) E13.5 brain sections stained for Isolectin B4. Blood vessels were more abundant and larger in *ko* than in controls. Scale bar is 1 mm in (**a**) and 200 µm in (**b**).

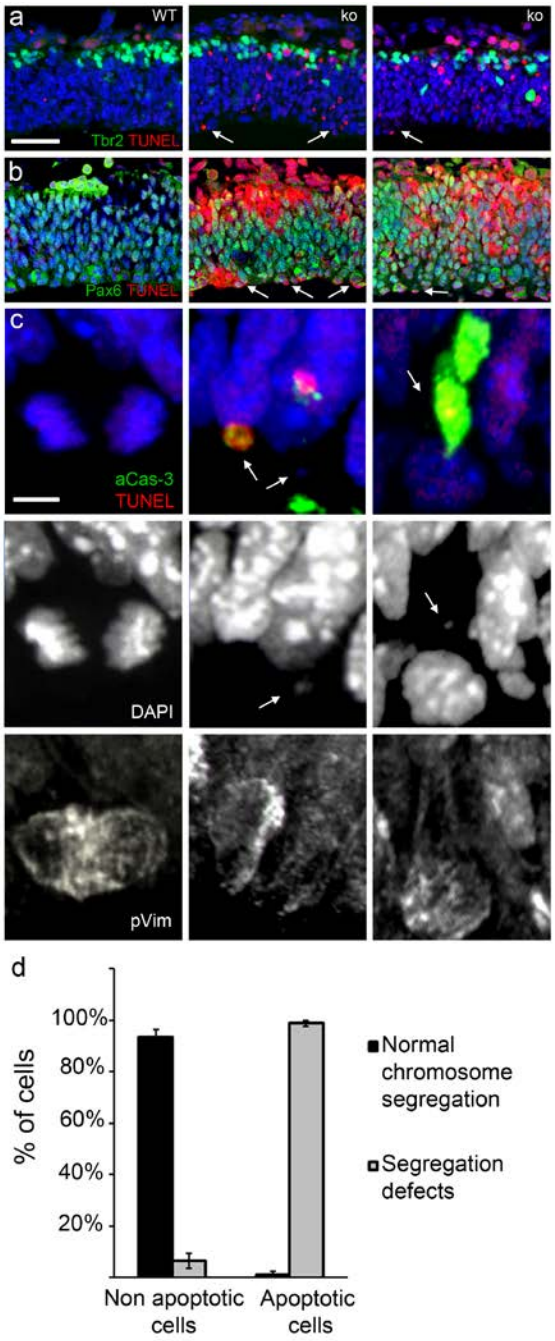

Supplementary Figure 3 Characterization of apoptotic cells

Cortical walls from E10.5 embryos were co-stained for TUNEL and Tbr2 (**a**), Pax6 (**b**), and aCas3 (**c**). TUNEL-stained cells in the vicinity of the VZ edge are shown by arrows in (**a**) and (**b**). (**c**) DAPI staining highlighting the presence of micronuclei and lagging chromosomes (arrows) in TUNEL and aCas-3 positive mitotic ($pVim^+$) cells. Scale bars are 50 µm in (**a**,**b**) and 5 µm in (**c**). (**d**) Percentage of cells with mitotic errors in normal versus apoptotic cell populations. Error bars represent Standard Error of the Mean (SEM).

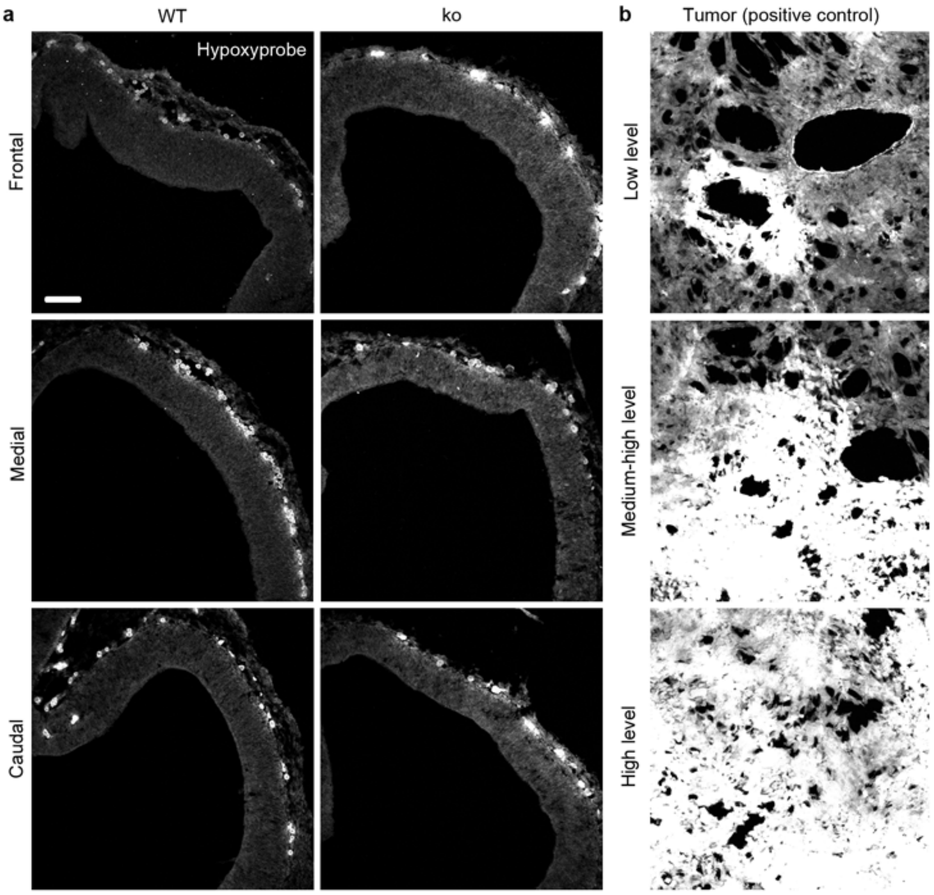

Supplementary Figure 4 No hypoxia in the dorsal telencephalon of *Diaph3* mutants.

(**a**) E10.5 cortical sections were stained with an anti-Hypoxyprobe antibody. No difference was detected between WT and *ko* littermates. (**b**) Tumor xenografts were used as positive control for Hypoxyprobe staining. Different degrees of hypoxia are shown. Scale bar is 50 µm.

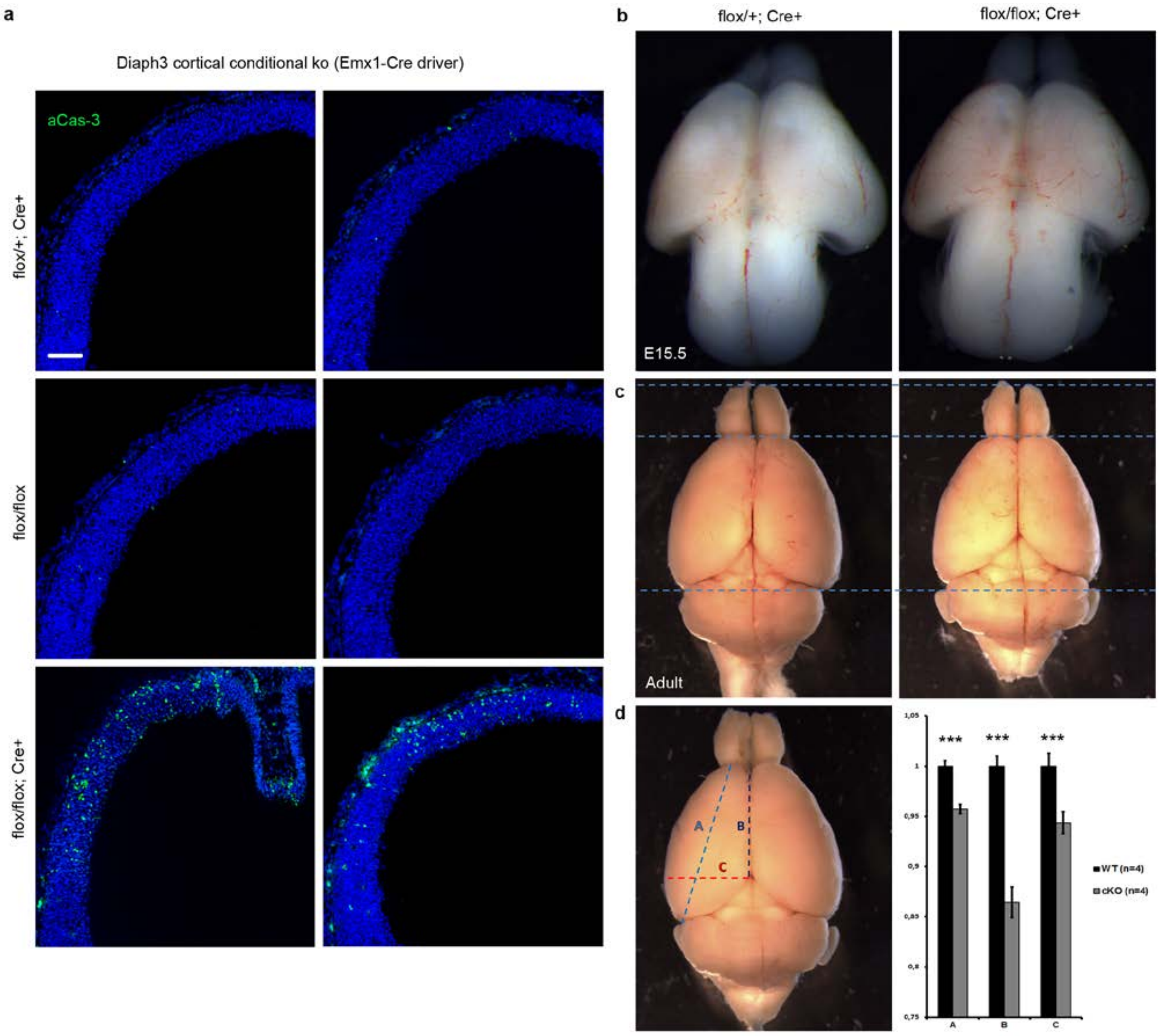

Supplementary Figure 5 Inactivation of *Diaph3* in progenitors causes microcephaly.

(**a**) Coronal sections from conditional mutants and controls were stained with aCas3 (green). Numerous apoptotic cells were scattered through the cortical wall of *Diaph3*$^{flox/flox}$;*Emx1-Cre* mice, whereas no positive cells were observed in *Diaph3*$^{flox/+}$;*Emx1-Cre*, or *Diaph3*$^{flox/flox}$ littermates. Scale bar is 50 µm. (**b–d**) Reduction of cortical size in *Diaph3* conditional *ko* mice at E15.5 (**b**) and in the adult (**c**). (**d**) Quantification of the cortical size in *Diaph3* conditional *ko* mice as measured along three axes (A, B, and C, left) and normalized to control littermates. Differences were statistically significant (axis A: $P < 0.0001$; axis B: $P = 0.0003$; axis C: $P = 0.005$; $n = 4$ brains for each genotype, Student's *t*-test). Error bars represent Standard Error of the Mean (SEM).

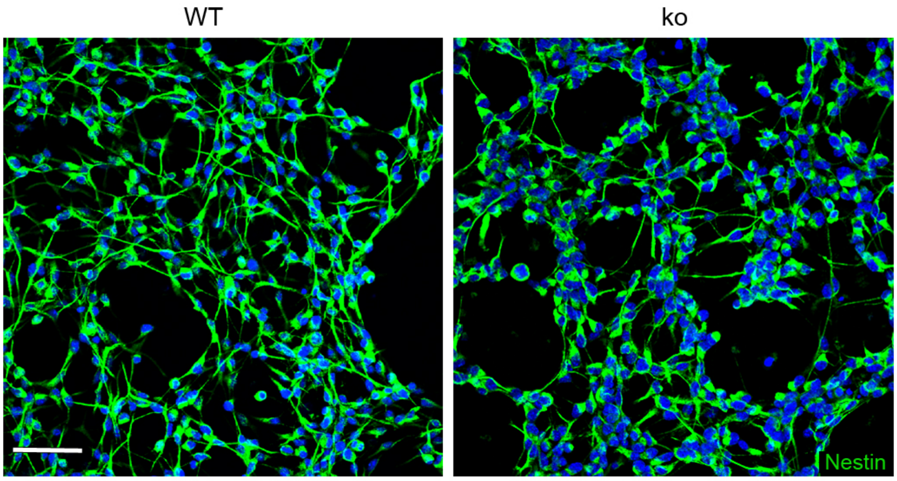

Supplementary Figure 6 Neuroepithelial cells maintain neural identity *in vitro.*

Nestin immunostaining (green) of dissociated neurospheres from wild-type (WT) and mutant (ko) at passage 8 confirms their neural identity. Nuclei were counterstained with DAPI. Scale bar is 50 µm.

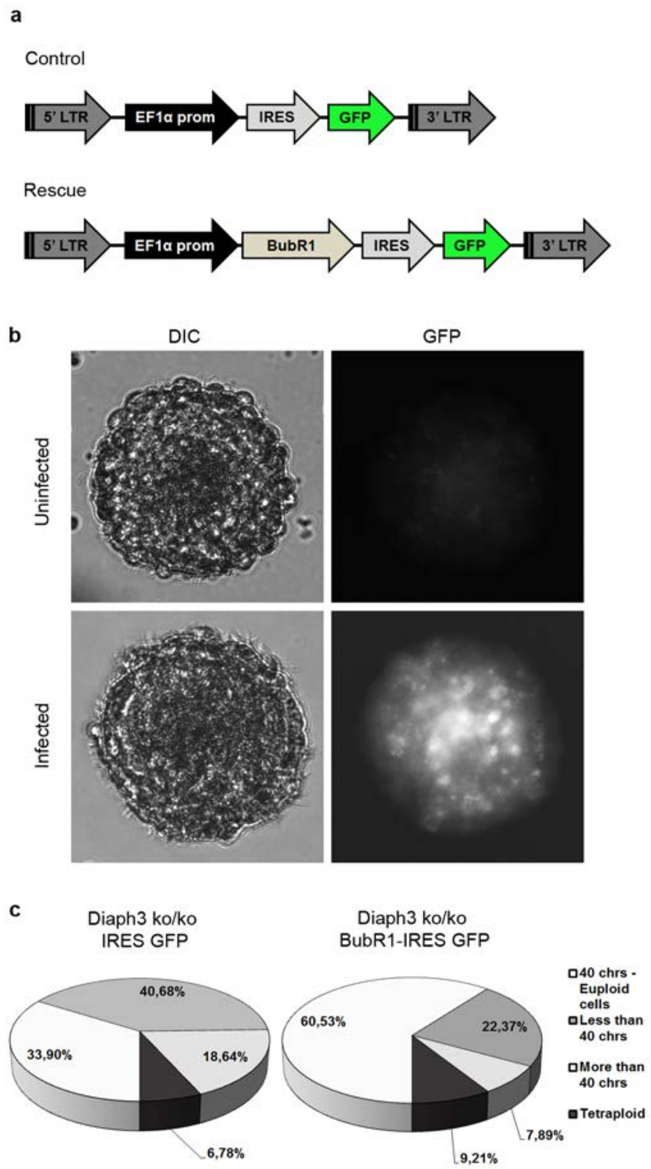

Supplementary Figure 7 Partial rescue of aneuploidy by BubR1 overexpression.

Diaph3 ko dissociated neurospheres were infected with either IRES-GFP (Control) or BubR1-IRES-GFP (Rescue) lentiviral vectors (**a**). Fluorescent neurospheres (**b**) were amplified and processed for karyotype analysis (**c**). The number of euploid cells was almost twofold higher in BubR1-infected than in GFP-infected spheres (60,5% *versus* 33,9%, n = 76 for BubR1- and 59 for GFP-infected spheres).

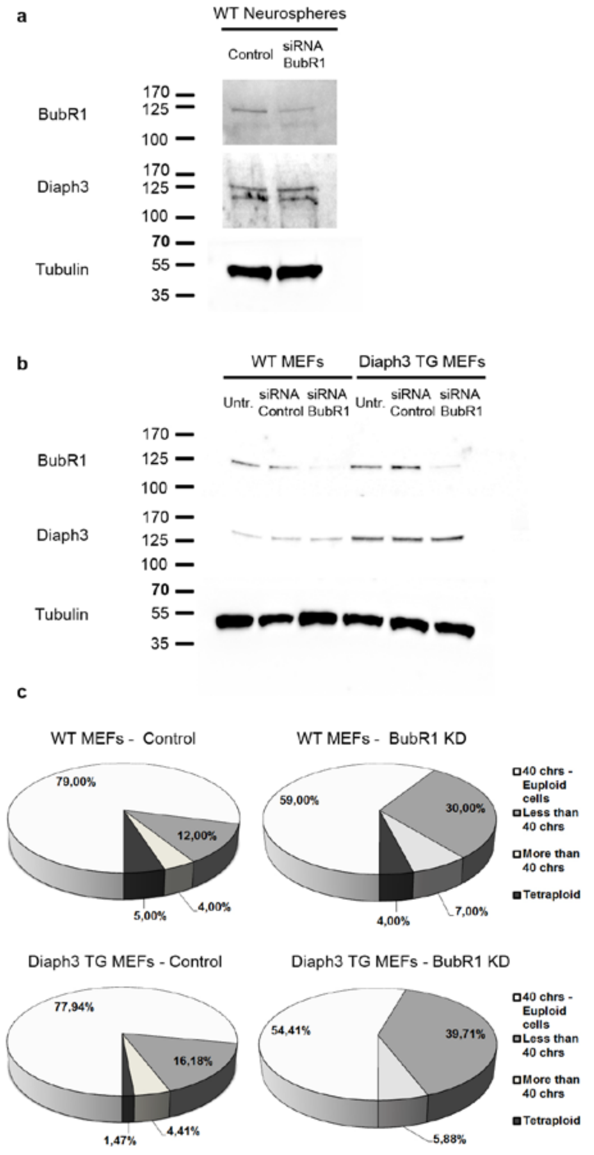

Supplementary Figure 8: BubR1 downregulation has no effect on Diaph3 protein levels. Western blot analysis showing the downregulation of BubR1 in neurospheres (**a**), and in MEFs from control or Diaph3 transgenic (TG) mice (**b**). Note that Diaph3 levels were not affected upon BubR1 downregulation. (**c**) Karyotype analysis of control (top) and Diaph3-overexpressing (TG) (bottom) MEFs upon BubR1 knockdown (KD). No significant difference was observed between the two genotypes suggesting that the overexpression of Diaph3 could not rescue the aneuploidy induced upon BubR1 knockdown (*n* = 73 WT, 86 WT-BubRI KD, 91 Diaph3 TG, and 78 Diaph3 TG-BubR1 KD metaphases).

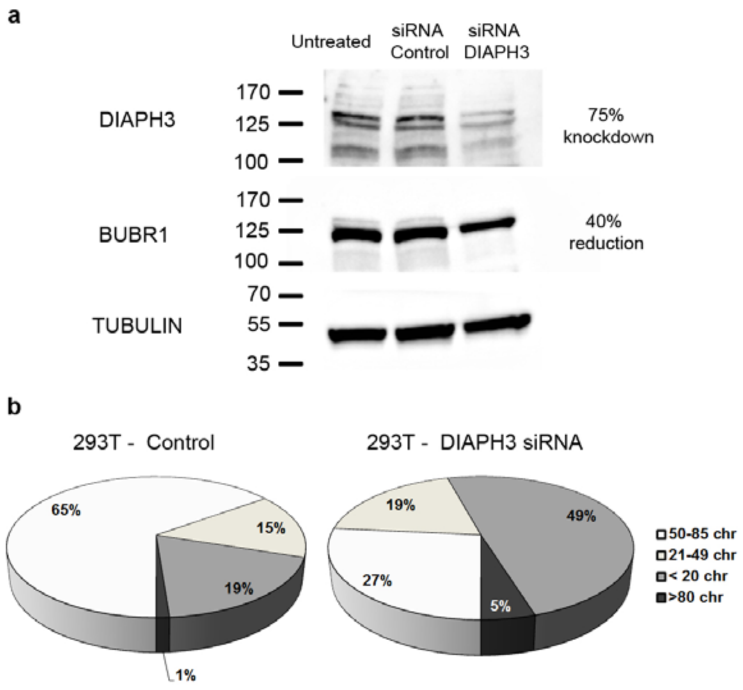

Supplementary Figure 9 Downregulation of DIAPH3 increases aneuploidy.

(**a**) Western blot analysis showing a ~75% decrease in the levels of DIAPH3 in HEK-293T cells treated with DIAPH3 siRNA (versus cells treated with siRNA control). A concomitant (~40%) reduction of BUBR1 was detected. **(b)** Upon DIAPH3 knock-down, the percentage of hypotriploid metaphases (number of chromosomes ranging from 50 to 85) decreased dramatically (65% for controls *versus* 27% for knockdown cells), whereas the proportion of metaphase spreads with very low number of chromosomes (less than 20) increased to 49% in knockdown *versus* 19% in control cells (*n* = 112 control and 105 Diaph3 KD metaphases).

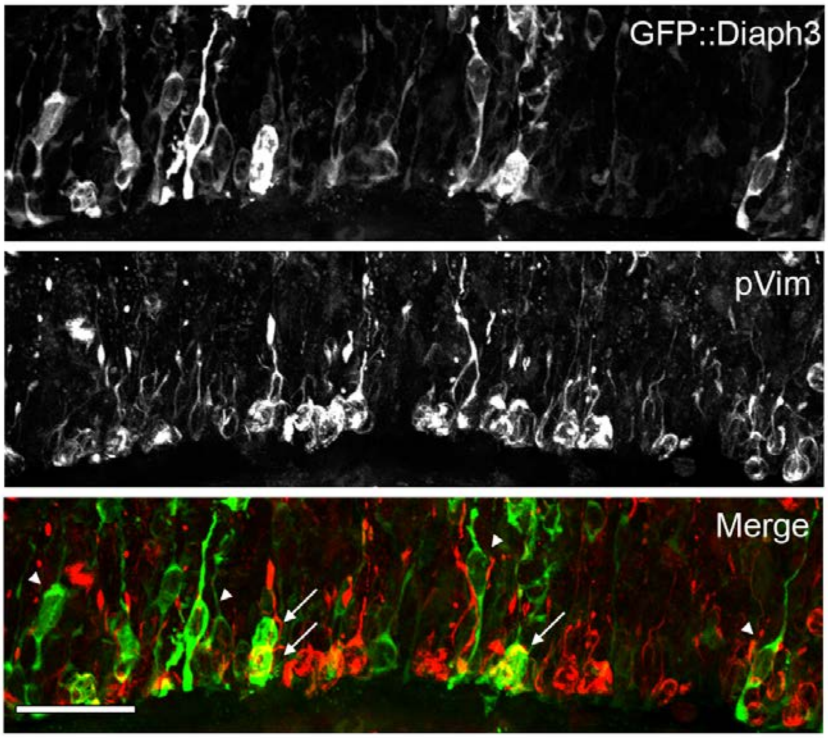

Supplementary Figure 10 Cellular localization of Diaph3.

Cortical sections from E14.5 embryos that were electroporated *in utero* at E13.5, with a plasmid coding for GFP::Diaph3 fusion protein. In non-dividing cells (arrowheads), the GFP::Diaph3 was perinuclear and associated with apical and basal processes. In dividing cells (arrows) the signal was more diffusely distributed around the chromatin. Scale bar is 50 µm.

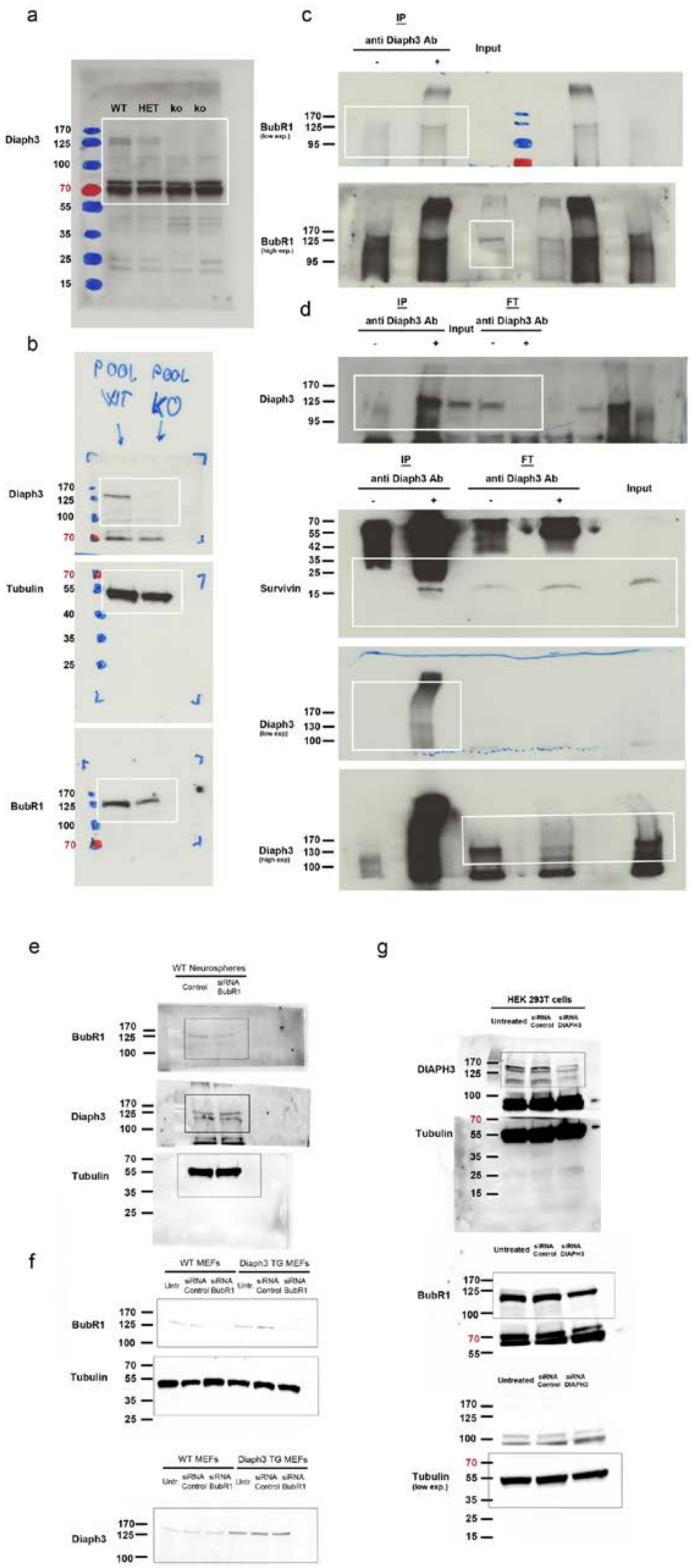

Supplementary Figure 11 Scans of original Western blots shown in Fig. 1f (**a**); Fig. 5h (**b**); Fig. 5j (**c**); Fig. 6 a (**d**); Supplementary Fig. 8a (**e**); Supplementary Fig. 8b (**f**); and Supplementary Fig. 9a (**g**)

Genotype (n)	Examined cells	Euploid cells	Aneuploid cells	Number of chromosomes in aneuploid cells											
				<35	35	36	37	38	39	41	42	43	44	45-49	80
Diaph3$^{+/+}$ (2)<br>*Diaph3*$^{ko/+}$ (2)	129	116	13	0	0	0	0	3	5	4	1	0	0	0	0
Diaph3$^{ko/ko}$ (4)	161	57	104	8	9	5	4	8	25	20	9	3	1	6	6

Supplementary Table 1 Chromosome counts in neurospheres of indicated genotypes.
